# Dietary isoflavones alter regulatory behaviors, metabolic hormones and neuroendocrine function in Long-Evans male rats

**DOI:** 10.1186/1743-7075-1-16

**Published:** 2004-12-23

**Authors:** Edwin D Lephart, James P Porter, Trent D Lund, Lihong Bu, Kenneth DR Setchell, Gina Ramoz, William R Crowley

**Affiliations:** 1 Department of Physiology and Developmental Biology, Brigham Young University, Provo, UT, USA; 2 The Neuroscience Center, Brigham Young University, Provo, UT, USA; 3 Biomedical Sciences, Colorado State University, Fort Collins, CO, USA; 4 Department of Pediatrics, Children's Hospital Medical Center, Cincinnati, OH, USA; 5 Pharmacology & Toxicology, University of Utah, College of Pharmacy, Salt Lake City, UT, USA

## Abstract

**Background:**

Phytoestrogens derived from soy foods (or isoflavones) have received prevalent usage due to their 'health benefits' of decreasing: a) age-related diseases, b) hormone-dependent cancers and c) postmenopausal symptoms. However, little is known about the influence of dietary phytoestrogens on regulatory behaviors, such as food and water intake, metabolic hormones and neuroendocrine parameters. This study examined important hormonal and metabolic health issues by testing the hypotheses that dietary soy-derived isoflavones influence: 1) body weight and adipose deposition, 2) food and water intake, 3) metabolic hormones (i.e., leptin, insulin, T3 and glucose levels), 4) brain neuropeptide Y (NPY) levels, 5) heat production [in brown adipose tissue (BAT) quantifying uncoupling protein (UCP-1) mRNA levels] and 6) core body temperature.

**Methods:**

This was accomplished by conducting longitudinal studies where male Long-Evans rats were exposed (from conception to time of testing or tissue collection) to a diet rich in isoflavones (at 600 micrograms/gram of diet or 600 ppm) vs. a diet low in isoflavones (at approximately 10–15 micrograms/gram of diet or 10–15 ppm). Body, white adipose tissue and food intake were measured in grams and water intake in milliliters. The hormones (leptin, insulin, T3, glucose and NPY) were quantified by radioimmunoassays (RIA). BAT UCP-1 mRNA levels were quantified by PCR and polyacrylamide gel electrophoresis while core body temperatures were recorded by radio telemetry. The data were tested by analysis of variance (ANOVA) (or where appropriate by repeated measures).

**Results:**

Body and adipose tissue weights were decreased in Phyto-600 vs. Phyto-free fed rats. Food and water intake was greater in Phyto-600 animals, that displayed higher hypothalamic (NPY) concentrations, but lower plasma leptin and insulin levels, vs. Phyto-free fed males. Higher thyroid levels (and a tendency for higher glucose levels) and increased uncoupling protein (UCP-1) mRNA levels in brown adipose tissue (BAT) were seen in Phyto-600 fed males. However, decreased core body temperature was recorded in these same animals compared to Phyto-free fed animals.

**Conclusions:**

This study demonstrates that consumption of a soy-based (isoflavone-rich) diet, significantly alters several parameters involved in maintaining body homeostatic balance, energy expenditure, feeding behavior, hormonal, metabolic and neuroendocrine function in male rats.

## Background

Some phytochemicals are considered to be endocrine disrupters that mimic or modulate the physiological effects of steroid hormones, especially that of estrogens [1,2]. Of all estrogenic endocrine disrupters examined thus far, phytoestrogens have been extensively studied [1-6].

Phytoestrogens represent hundreds of molecules possessing non-steroidal, diphenolic structures found in many plants (e.g. fruits, vegetables, legumes, whole-grain and especially soy food products) that have similar chemical and structural properties to those of estrogens [1-4]. There are three main classifications of phytoestrogens: 1) isoflavones (derived principally from soybeans), 2) lignans (found in flaxseed in large quantities) and 3) coumestans (derived from sprouting plants like alfalfa) [2-6].

Of these three main classifications, human consumption of isoflavones has the largest impact due to its availability and variety in food products containing soy. Furthermore, the phytoestrogens principally derived from soy foods have received prevalent usage due to their 'health benefits' of decreasing: a) age-related diseases (cardiovascular & osteoporosis), b) hormone-dependent cancers (e.g. breast & prostate) and c) postmenopausal symptoms [2-6]. However, little is known about the influence of dietary (soy-derived) phytoestrogens on neuroendocrine, hormone and metabolic parameters. In spite of this fact, the Food and Drug Administration (FDA) in the United States in October of 1999 authorized the use of-on food labels- the health claim that: soy protein can reduce the risk of coronary heart disease by lowering blood cholesterol levels (when included in a diet low in saturated fat and cholesterol) [5].

The purpose of this study was to examine, in a comprehensive manner, important hormonal and metabolic health issues by testing the hypotheses that dietary soy-derived phytoestrogens influence: 1) body weight and adipose deposition, 2) food and water intake, 3) metabolic hormones (i.e., leptin, insulin, T3 and glucose levels), 4) brain neuropeptide Y (NPY) levels, 5) heat production [in brown adipose tissue (BAT) quantifying uncoupling protein (UCP-1) mRNA levels] and 6) core body temperature. This was accomplished by conducting longitudinal studies where male Long-Evans rats were exposed (from conception to time of testing or tissue collection) to a diet rich in phytoestrogens vs. a diet low in phytoestrogens.

## Methods

### Animals

Long-Evans male and female rats [10 per sex at 50 days old] were purchased from Charles River Laboratories (Wilmington, MA, USA) for breeding. These animals were caged individually and housed in the Brigham Young University Bio-Ag vivarium and maintained on an 11-hour dark 13-hour light schedule (lights on 0600–1900). The animals and methods of this study were approved by the institute of animal care and use committee (IACUC) at Brigham Young University (BYU).

### Treatment-Diets

**Figure 1:**
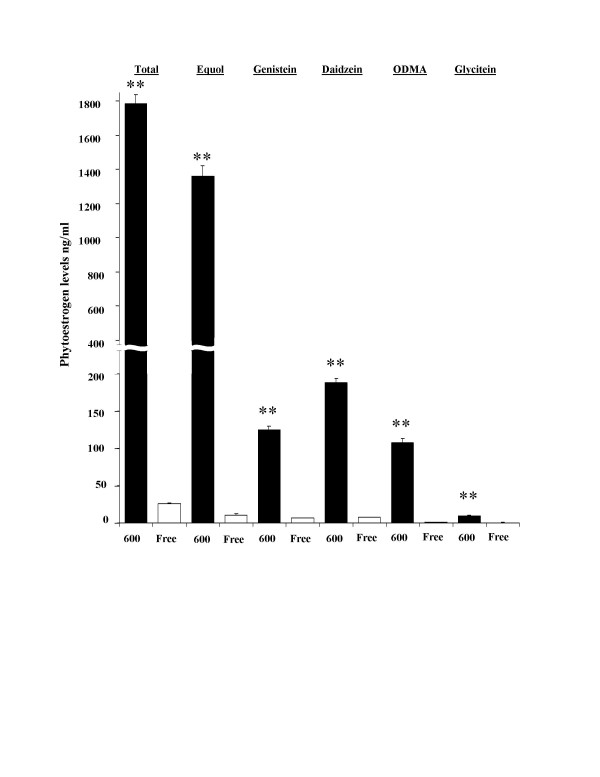
Serum Isoflavone Levels in Phytoestrogen-rich (Phyto-600) vs. Phytoesterogen-low (Phyto-Free) Fed Male Rats at 75-Days of Age. For each phytoestrogen measured the Phyto-600 animals displayed significantly higher (** p < 0.01) isoflavone levels compared to Phyto-free fed rats. ODMA = O-desmethylangolensin. Equol levels in Phyto-600 animals accounted for approximately 78% of the total phytoestrogen levels.

Upon arrival all animals were allowed ad libitum access to either a commercially available diet with high phytoestrogen levels (Harlan Teklad Rodent Diet 8604, Madison, WI, USA) containing 600 micrograms of phytoestrogens/gram of diet [or specifically this diet is high in isoflavones, 600 parts per million or ppm]; referred to hereafter as the Phyto-600 diet, or a custom phytoestrogen-free diet; referred to hereafter as the Phyto-free diet, obtained from Ziegler Bros. (Gardner, PA, USA) and water [7]. In the Phyto-free diet, the phytoestrogen concentrations were below the detectable limits of HPLC analysis [7]. The content and nutrient composition of these diets is described in detail elsewhere [7]. The diets were balanced and matched for equivalent percentage content of protein, carbohydrate, fat, amino acids, vitamins and minerals, etc. [7]. Circulating phytoestrogen serum levels from rats maintained on these diets (lifelong) have been reported previously by our laboratory using GC/MS analysis [7]. The animals were time mated within their respective diets so that the offspring of these pairings would be exposed solely to either the Phyto-600 or Phyto-free diet. Parameters were measured and/or the male rats were sacrificed and blood and tissues collected mainly at 33, 55 or 75 days of age; other ages were tested where indicated. Serum was prepared and stored at -20°C until assayed for metabolic hormones. For this study serum isoflavone levels are shown in Figure 1 from animals at 75 days of age. Male rats were only examined in this study since the influence of the estrous cycle on several of the measured parameters is unknown.

### Weight measurements

Body weights and food intake were measured on a Mettler 1200 balance [in grams (g) ± 1 g; St. Louis, MO, USA], white and brown adipose tissue and prostate weights were measured on a Sartorious balance [in milligrams (mg) ± 1 mg; Brinkman Inst. Co., Westbury, NY, USA]. Water intake was measured in drinking tubes [in milliliters (ml) ± 1 ml]. White adipose tissue (WAT) was dissected inferior to the kidneys and superior to the testes in the abdominoplevic cavity (representing a majority of intra-abdominal WAT) and then weighed in grams ± 0.01 g. Brown adipose tissue was dissected from between the scapular blades (inter-scapular region) and weighed in milligrams (mg) ± 1 mg.

### Metabolic Hormones

Serum leptin and insulin levels were determined by kits purchased from Linco Res. Inc. (St. Charles, MO, USA) [from arterial blood samples of 33 and 55 day-old male animals and venous blood samples collected from 75 day-old rats. This was due to exhausting the arterial supplies from the available blood samples for other assays and thus venous blood was assayed at 75 days of age]. Serum thyroid (T3) levels were assayed by a kit purchased from Diagnostic Systems Labs. Inc. (Webster, TX, USA) and glucose levels were detected by a kit (#510) purchased from Sigma Chem. Co. (St. Louis, MO, USA).

### Hypothalamic NPY Levels

Subsequent to blood collection (above), after the animals were sacrificed, brains were removed rapidly, frozen on dry ice and then stored at -80°C until microdissection. Coronal slices 300 μm thick were sectioned on a microtome cryostat. The paraventricular nucleus, arcuate nucleus and median eminence regions of the hypothalamus were microdissected by punch technique and homogenized in 100 μl of 0.1 M HCl. Tissue protein was determined by the Lowry method [8] and NPY was measured using a solid-phase radioimmunoassay in Protein G-coated 96-well plates, as described previously [9]. The NPY antiserum was used at a final concentration of 1:16,000. The sensitivity of the assay is 0.2 pg, with an intra-assay coefficient of variation of 8 %. All samples were run in duplicate in the same assay to avoid inter-assay variation.

### Body temperature

Body temperature was monitored by radio telemetry by implanting a very small electronic chip [under the skin above the left thoracic cavity near the heart] that measured and transmitted core body temperature (± 0.1°C) to a notebook-sensor monitor (BioMedic Data Systems Inc., Seaford, DE, USA) within 2 seconds and repeated measurements were made throughout the day and/or the duration of the experiments.

### Body Heat Production

Uncoupling protein (UCP-1) mRNA levels were determined in brown adipose tissue (BAT) collected from the interscapular region of each male rat. The BATs were homogenized in Trizol reagent (Invitrogen, Carlsbad, CA, USA) and total RNA was extracted. Two micrograms (2 μg) of total RNA were reverse transcribed (RT) for 60 min at 42°C using Superscript II (Invitrogen, Carlsbad, CA, USA) (200 U). Each 20 μl reaction contained 0.1 M DTT (2 μl), 10 mM dNTP mix (1 μl), 10X PCR buffer (2 μl), random decamers (0.4 μl), and RNaseOUT (Invitrogen) (40 U). A duplex PCR reaction was then performed on each RT product, with 18S rRNA serving as the internal control. Each 50 μl reaction contained the RT product (2 μl), UCP-1 primers (2 μl), 18S primers [2 μl of 3:7 ratio of 18S primer to18S competimer (Ambion, Austin, TX, USA)], 10X PCR buffer (5 μl), 10 mM dNTP mix (0.625 μl), ^32^P-dCTP (0.1–0.15 μl), and Jumpstart Taq polymerase (Sigma Chem. Co., St. Louis, MO, USA) (1 U). Each tube was then subjected to the following protocol: 95°C for 5 min, 20 cycles of 94°C for 30 sec, 60°C for 30 sec, 72°C for 45 sec, followed by 72°C for a final 10 min. interval. With this profile, the UCP-1 and 18S fragments were amplified within the linear range (20 cycles for UCP-1). The primers for UCP-1 were GTGAAGGTCAGAATGCAAGC (sense) and AGGGCCCCCTTCATGAGGTC (antisense), the resultant fragment was 197 bp. The sequence of the UCP-1 fragment was verified by the DNA Sequencing Center at BYU. The PCR products were then subjected to non-denaturing polyacrylamide gel electrophoresis and the gels were exposed to autoradiographic film. Optical density (O.D.) of each band was determined using the NIH imaging system (Version 1.61). For each sample the O.D. ratio UCP:18S was determined. Each RT-PCR protocol was repeated and O.D. ratio values averaged over at least two runs.

### Statistical Analysis

All data are presented as the mean ± SEM with p < 0.05 deemed significant. The data were tested by analysis of variance (ANOVA) (or where appropriate by repeated measures), followed by pairwise comparisons (via Neuman-Keuls analysis) to detect significant differences between the diet treatment groups (p < 0.05).

## Results

### Body Weight, White Adipose Tissue Weight and Food/Water Intake

**Figure 2:**
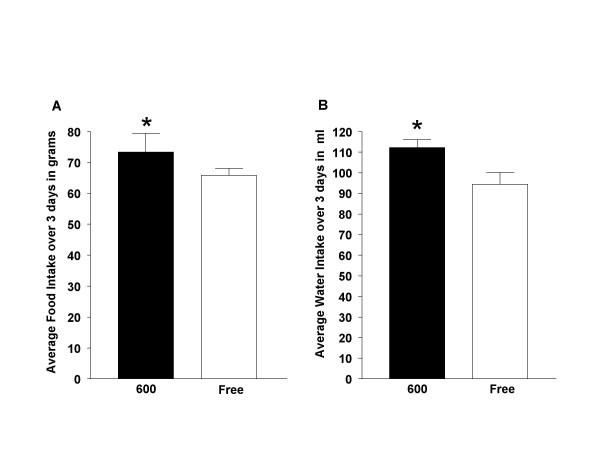
Effects of Dietary Phytoestrogens on Food and Water Intake in 75 Day-Old Male Long-Evans Rats. Males fed a phytoestrogen-rich (600) diet displayed significantly greater (* p < 0.05) food (A) and water intake (B) compared to males fed a phytoestrogen-free (Free) diet. The average food and water intake represents the volumes consumed over 3 consecutive days.

When food and water intake was measured in young adult animals, surprisingly the Phyto-600 fed males displayed slight but significantly higher food (Figure 2A) and water (Figure 2B) consumption compared to Phyto-free fed males [for food intake: Phyto-600 = 24.3 vs. Phyto-free = 21.7 grams/day (p < 0.05) and for water intake: Phyto-600 = 37.7 vs. Phyto-free = 31.2 ml/day (p < 0.05).

**Figure 3:**
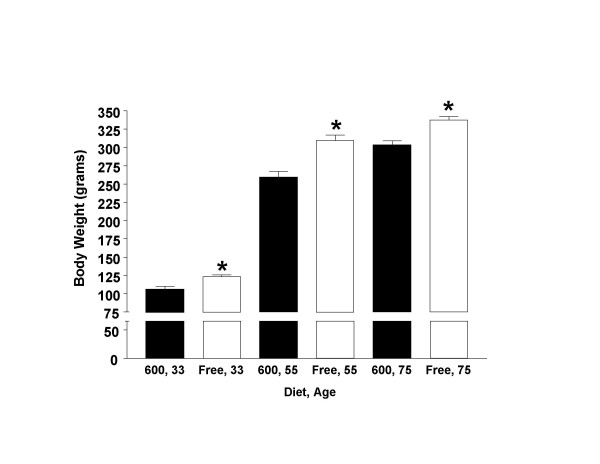
Effects of Dietary Phytoestrogens on Body Weight in Male Long-Evans Rats fed either a phytoestrogen-rich (600) or a phytoestrogen-free (Free) diet. At 33, 55 and 75 days-old Free-fed male body weights (* p < 0.05) were significantly greater compared to 600-fed male values.

The effects of dietary phytoestrogens on body weights in pre-, early adult and young adult age male rats are shown in Figure 3. At every age examined (i.e., 33, 55 and 75 days old), males exposed to the Phyto-free diet displayed significantly higher body weights (around 10–15%) compared to animals fed the Phyto-600 diet.

**Figure 4:**
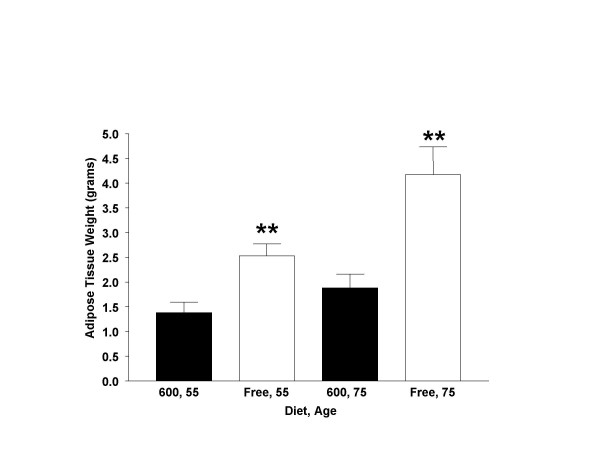
Effects of Dietary Phytoestrogens on White Adipose Tissue in Male Long-Evans Rats fed either a phytoestrogen-rich (600) or a phytoestrogen-free (Free) diet. At 55 and 75 days post-birth white adipose tissue weight was significantly greater in Free-fed males (** p < 0.01) compared to 600-fed male values.

White adipose tissue (WAT) weights were not measured in 33 day-old animals, since relatively little fat deposition was observed in the abdominopelvic cavity (especially around the reproductive structures) at this age. However, at 55 and 75-days of age, males fed the Phyto-free diet displayed significantly higher white adipose tissue weights (approximately 50% greater) compared to Phyto-600 values (Figure 4).

### Circulating Leptin, Insulin, Glucose and Brain NPY Levels

**Figure 5:**
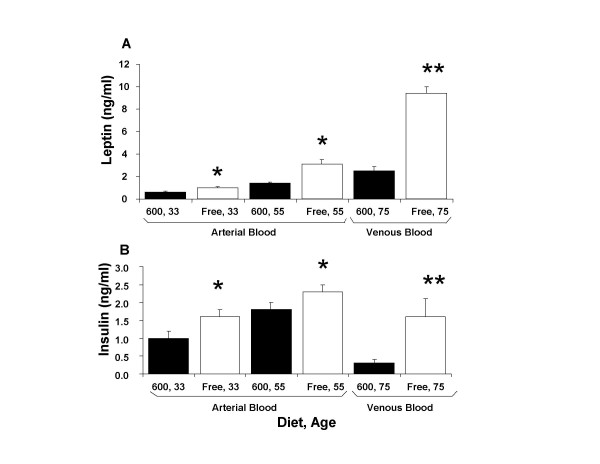
Plasma Leptin (A) and Insulin (B) Levels from 33, 55 or 75 day-old Male Long-Evans Rats fed either a phytoestrogen-rich (Phyto-600) or a phytoestrogen-free (Phyto-Free) diet. At 33, 55 and 75 days of age, males fed the phytoestrogen-free (Free) diets displayed significantly higher leptin and insulin levels (* p < 0.05, ** p < 0.01) compared to males fed the Phyto-600 (600) diet.

In 33, 55 and 75 day-old male rats, circulating leptin and insulin levels were within the normal ranges (as described by the vendor's assay kit values), however, at each age males fed the Phyto-free diet displayed significantly higher leptin (Figure 5A) and insulin (Figure 5B) levels compared to Phyto-600 values. Notably, the leptin levels significantly increased with age that corresponded with significantly higher white adipose tissue deposition seen in these animals.

From the animals collected on 33, 55 and 75 days of age not enough serum was left after other assays were performed to quantify glucose levels on these days. However, circulating glucose levels were assayed in non-fasting 65, 80 or 110 day-old animals, Phyto-600 fed males displayed slightly higher values (that were not significantly different) compared to Phyto-free fed males [age 65 days old- Phyto-600 = 113.5 (± 4.4) vs. Phyto free = 93.5 (± 9.0) mg/dl, n = 8 per group; age 80 days old- Phyto-600 = 137.2 (± 3.8) vs. Phyto-free = 122.5 (± 3.9) mg/dl, n = 10 per group; age 110 days old- Phyto-600 = 123.5 (± 5.0) vs. Phyto-free = 113.8 (± 3.6) mg/dl, n = 5 per group, (mean ± SEM), data not shown graphically].

**Figure 6:**
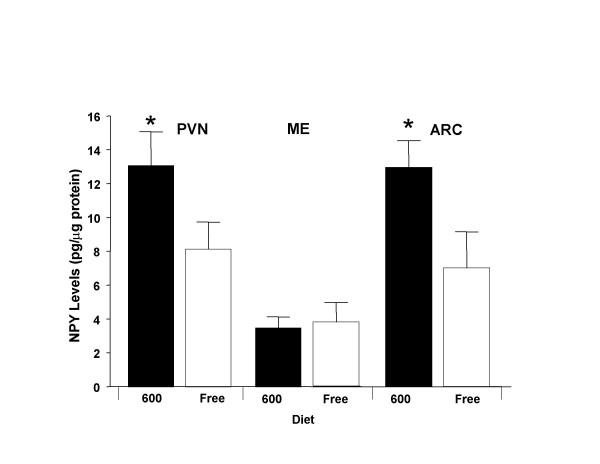
Dietary Phytoestrogens Influence on Brain NPY Levels in 75 day-old Male Long-Evans Rats. In the paraventricular (PVN) and arcuate (ARC) nucleus, males fed the Phyto-600 (600) diet displayed significantly greater NPY levels (* p < 0.05) compared to males fed the Phyto-Free (Free) diet. In the median eminence (ME) no significant differences were observed between male rats fed 600 vs. the Free diet.

Since leptin plays an important role in regulating brain NPY levels that in turn influences food/water intake, NPY levels were determined in three hypothalamic regions [i.e., the periventricular nucleus (PVN), median eminence (ME) and the arcuate nucleus (ARC)] in 75 day-old males exposed to the diet treatments. In the PVN and ARC (but not the ME) NPY levels were significantly higher (by approximately 40 %) in Phyto-600 fed males vs. the Phyto-free male values (Figure 6).

### Circulating Thyroid (T3), UCP-1 mRNA Levels and Core Body Temperature

In non-fasting young adult rats at 65 and 110 days of age, circulating thyroid (T3) levels were determined from venous blood samples. Phyto-600 fed males displayed significantly higher T3 levels compared to Phyto-free fed values [age 65 days old- Phyto-600 = 2.4 ± 0.2 vs. Phyto-free = 1.5 ± 0.4 pg/ml (± SEM), n = 8 per diet treatment; age 110 days old- Phyto-600 = 1.9 ± 0.4 vs. Phyto-free = 0.8 ± 0.3 pg/ml, n = 5 per group, (mean ± SEM) data not shown graphically].

**Figure 7:**
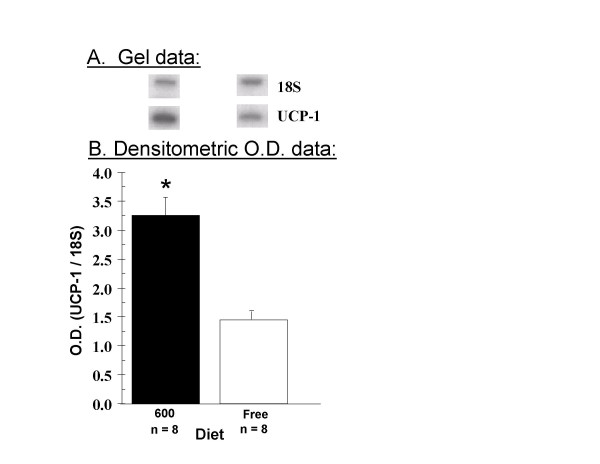
Dietary Phytoestrogens Influence on Uncoupling Protein-1 (UCP-1) mRNA Levels in Brown Adipose Tissue (BAT) from males fed either a phytoestrogen-rich (Phyto-600) or a phytoestrogen-free (Phyto-free) diet. For each sample, the optical density (O.D.) signal ratio of BAT UCP-1 mRNA abundance to ribosomal 18S RNA intensity was determined. The results represent the mean O.D. values and S.E.M. of 8 independent samples by diet treatment of at least two runs of the RT-PCR protocol. Males fed the Phyto-600 diet expressed significantly higher BAT UCP-1 mRNA levels (* p < 0.05) compared to Phyto-free values.

The effects of dietary phytoestrogens on uncoupling protein-1 (UCP-1) mRNA levels in brown adipose tissue (BAT) from 75 day-old animals is shown in Figure 7. Phyto-600 fed males displayed significantly higher (≈ 2-fold) UCP-1 mRNA levels in BAT compared to Phyto-free values. Notably, the BAT weights of Phyto-600 animals were significantly less (by approximately 1/3) to that of Phyto-600 males (Phyto-600 = 293.6 ± 22.1 vs. Phyto-free = 437.5 ± 25.9 mg (mean ± SEM), n = 8 per group.

**Figure 8:**
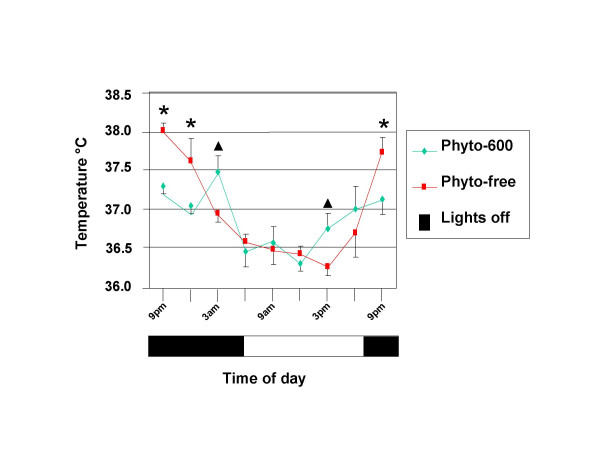
Dietary Phytoestrogens Influence on Core Body Temperature in 75 day-old rats. In general, during the dark phase of the light cycle, Phyto-free fed males displayed significantly higher core body temperatures (* p < 0.05) compared to Phyto-600 fed values. However, near the end of the dark (at 3 am) and light (at 3 pm) phase of the dark/light cycle Phyto-600 fed males displayed significantly higher core body temperatures (▲ p < 0.05) compared to Phyto-free values.

Finally, when core body temperatures were recorded during a 24-hour interval, Phyto-free fed males displayed, in general, slight but significantly higher values compared to Phyto-600 animals during the dark phase of the light/dark cycle when rodents are most active (Figure 8). However, during one time point during the dark cycle (3 am) and one time point during the light phase of the cycle (3 pm) Phyto-600 males displayed slight but significantly higher core body temperatures vs. Phyto-free values.

## Discussion

Estrogen is known to play a dual role in regulating body weight, food intake and adipose tissue deposition. On the one hand, estrogens decrease food intake, increase locomotor activity and hence decrease body weight [10,11]. However, adipose tissue deposition increases with puberty and early pregnancy in women, suggesting that estrogens influence body fat accumulation [12]. Additionally, in aging, estrogens promote adipose deposition and insulin resistance [13]. Conversely, results from aromatase, FSH and ER-knockout studies indicate that estrogens regulate adiposity where the complete lack of estrogens or blocking estrogen hormone action increases adipose tissue deposition [14-18], whereas, estrogen replacement in these models decreases adiposity. Notably, in the present study, male rats fed the Phyto-600 diet displayed significantly decreased adipose tissue and body weights compared to Phyto-free fed animals. While there is not extensive data on phytoestrogens and metabolism, other investigators have reported that genistein, increases lipolysis and decreases lipogenesis in rodent adipocytes [19] by a tyrosine kinase independent mechanism and these estrogen mimics inhibit glucose uptake by altering membrane-associated glucose transporters [20,21]. Thus, our data suggests that dietary soy phytoestrogens significantly decrease: 1) body and adipose tissue weights and 2) circulating leptin and insulin levels (that correspond with adipose deposition) compared to Phyto-free fed animals, implying that the hormonal action of phytoestrogens is beneficial to body fat regulation. Recent studies imply that insulin helps to regulate leptin expression in humans [22] and estrogens appear to enhance the action of insulin [23,24]. This may account for the decreased incidence of obesity in Asian countries where isoflavone consumption is high compared to Western countries. Decreased adipose tissue deposition by decreasing lipogenesis and increasing lipolysis may help to prevent insulin resistance (by reducing body fat) and the estrogenic actions of dietary phytoestrogens may augment the efficiency of insulin.

It was previously observed in our laboratory that dietary phytoestrogens significantly alter food and water intake [7,25,26]. The differential effects of the Phyto-free vs. Phyto-600 diets observed in the present studies on hypothalamic NPY levels, circulating insulin and leptin concentrations and food intake are consistent with the well established interrelationships among these parameters. Thus, relative to animals maintained on the Phyto-free diet, food intake was significantly increased in animals fed the Phyto-600 diet. Phyto-600-fed rats also exhibited higher concentrations of NPY in the arcuate and paraventricular nuclei of the hypothalamus. It is well established that NPY neurons whose perikarya reside in arcuate nucleus and project to PVN comprise an extremely important orexigenic neural pathway [27]. It therefore appears likely that at least one factor contributing to the higher food intake in Phyto-600-fed rats is the increased levels of NPY in this system.

The present studies also suggest a mechanism that may underlie the diet-induced effects on NPY (i.e., plasma insulin and leptin concentrations were significantly reduced in the Phyto-600 fed rats, relative to the Phyto-free animals). A number of previous studies have demonstrated a reciprocal relationship between circulating insulin and leptin titers and NPY concentrations in PVN. Thus, experimentally-induced reductions in either insulin [28] or leptin [29] are associated with increased pre-proNPY messenger RNA expression in arcuate nucleus and increased NPY levels in PVN, and moreover, it has been proposed that reductions in insulin and leptin that occur physiologically, e.g., with food deprivation, provide an important signal to the NPY system to initiate feeding [27,29]. Hence, taken together, the present findings suggest that by reducing secretion of insulin and/or leptin, chronic consumption of the Phyto-600 diet results in up-regulation of the orexigenic NPY circuit in the hypothalamus, which in turn stimulates food intake (and water consumption, since rodents and humans display prandial characteristics).

**Figure 9:**
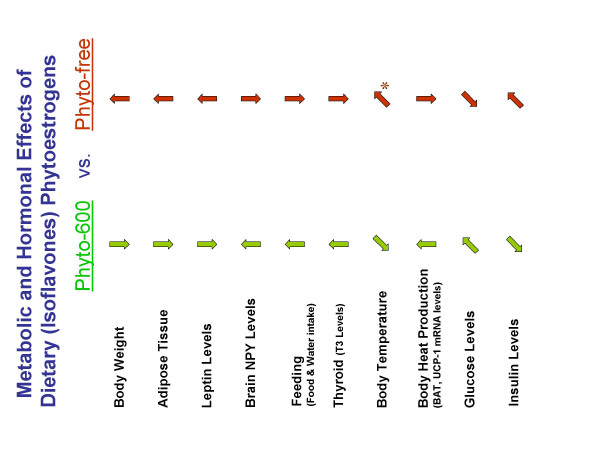
Summary of Dietary Phytoestrogen (Isoflavone) Influences on Metabolic and Hormonal Parameters. The left-hand column: Phyto-600 arrows are relative to the effects seen in Phyto-free fed animals-right-hand column [in other words, relative to one another]. * = general increase in body temperature compared to Phyto-600 values

While it is clear that thyroid hormone levels are influenced by estrogens where increases are seen in T3 and T4, presumably by increasing in the production of thyroid binding globulin in the liver [30], the published data examining thyroid function and hormone levels are problematic at best in the soy research field due to the history of soy food formulations, parameters examined and iodine deficiencies [6,31,32]. In agreement with more recent studies, our results demonstrate that circulating T3 levels increase with soy consumption [33], and "personal communication- Dr. David Baer-USDA". Furthermore, there appears to be a link between increased thyroid levels with soy consumption and cardiovascular protection in lowering serum cholesterol levels [6] and thyroid hormones along with estrogens protecting against osteoporosis [34]. However, in animals consuming the Phyto-600 diet (that displayed higher T3 levels) we observed a lower core body temperature compared to Phyto-free fed rats. In subsequent (unpublished) studies, we have consistently recorded slight (approximately 0.5°C) but significantly lower core body temperatures in Phyto-600 vs. Phyto-free fed rats during pregnancy. This suggests that the overall effect on body temperature via these estrogen mimics in the soy-rich diet may act primarily by increasing cutaneous vasodilation, thus decreasing core body temperature. Animal studies have shown that estrogens can act centrally (in the preoptic/anterior hypothalamus) or peripherally to regulate body temperature [35,36]. Support for this view is seen in humans where changes in skin blood flow via cutaneous vasodilation during the menstrual cycle and in hormone replacement therapy studies correspond with estrogen levels [36,37]. Also, one report showed that soy-derived phytoestrogens have a similar effect to our findings where ovariectomized rats fed a soy diet displayed an approximate 0.8°C decrease in skin temperature, whereas, estradiol treatment decreased temperature values by 1.4°C [38]. Finally, in association with temperature regulation, several studies have reported that soy consumption may be an effective therapy for relief of hot flushes in women [39]. Finally, the various metabolic parameters examined in a global fashion seem to suggest that declines in insulin and leptin levels are the dominant systemic regulators in regard to body weight, since overall the Phyto-600 animals weigh less compared to Phyto-free fed animals. However, the present findings also suggest that body temperature is reduced in Phyto-600 fed animals vs. Phyto-free fed animals and previous behavioral studies suggest that Phyto-600 animals exhibit more locomotor active vs. Phyto-free fed animals [7,50] (see summary Figure 9).

Uncoupling proteins (UCP-1 through UCP-5) are expressed in various tissues from many different species (mammals, birds, fish, insects and plants) that play important (but controversial) role(s) in the regulation of energy expenditure, or thermogenesis [40,41]. Uncoupling protein-1 is expressed mainly in BAT. When the influence of dietary phytoestrogens on UCP-1 mRNA levels in BAT was examined, Phyto-600 fed male rats, expressed significantly higher levels of the uncoupling protein (approximately 2-fold) compared to Phyto-free values (but BAT weights were significantly less in the Phyto-600 vs. Phyto-free fed males). To date, we are unaware of any studies that have investigated this aspect of soy consumption on thermogenesis. The decrease in BAT mass in Phyto-600 animals but increased expression of UCP-1 may represent a compensation mechanism for energy expenditure, and there are several neural inputs and hormonal factors that influence UCP-1 in BAT that make it difficult to differentiate the regulatory aspects of UCP-1 expression. For example, sympathetic denervation of inter-scapular BAT markedly reduced UCP-1 mRNA levels and estrogen, T3 and adrenergic agents [norepinephrine (NE)] stimulate UCP-1 expression in BAT [42,43]. In fact, it has been reported that T3 synergizes with NE to increase UCP-1 in BAT and stabilizes its mRNA transcripts [44]. These factors overlap with the changes seen in Phyto-600 fed vs. Phyto-free fed rats, in the present study, where T3 levels were increased and, presumably, along with the estrogenic influence of circulating isoflavones resulted in stimulating UCP-1 expression in BAT. Previously, we have not observed any significant alterations in circulating estradiol (or LH) levels in Phyto-600 vs. Phyto-free fed intact males [7]. Conversely, it has been reported that increases in hypothalamic NPY decrease UCP-1 [and reduces sympathetic outflow to BAT, but increases adipose tissue lipoprotein lipase activity] [30]. Also, plasma leptin levels are thought to stimulate UCP-1 in BAT [45,46], results opposite, in general, to that obtained in the present study. Based upon the obtained data sets, it is difficult to identify a common stimulatory or inhibitory pattern for the expression of UCP-1 in BAT of soy fed animals and especially define a functional role for the physiological properties associated with these UCPs in thermoregulation. Therefore, it is reasonable to speculate that multiple factors act collectively to regulate UCPs in BAT that in turn contribute to adaptive changes in body temperature.

## Conclusions

This study demonstrates that consumption of a widely used commercially available soy-based rodent diet, (i.e., the Phyto-600 diet rich in isoflavones), alters several hormonal, metabolic and neuroendocrine parameters involved in maintaining body homeostatic balance, energy expenditure and feeding behavior in male rats. Further research is warranted in examining the important aspects of the neuroendocrine and metabolic influences of dietary phytoestrogens via the consumption of soy in humans and laboratory animals. This is especially true when diet is usually not considered as an influencing factor in the experimental design [47-50].

## Abbreviations

neuropeptide Y (NPY), white adipose tissue (WAT), brown adipose tissue (BAT), uncoupling proteins (UCP), phytoestrogen-rich diet (Phyto-600), phytoestrogen free diet (Phyto-free), periventricular nucleus (PVN), median eminence (ME), arcuate nucleus (ARC), norepinephrine (NE), thyroid (T3, T4), National Institutes of Health (NIH), reverse transcriptase-polymerase chain reaction (RT-PCR), Food and Drug Administration (FDA), optical density (OD), luteinizing hormone (LH), follicle stimulating hormone (FSH), Brigham Young University (BYU), estrogen receptor (ER), high performance liquid chromatography (HPLC)

## Competing interests

The author(s) declare that they have no competing interests.

## Author's contributions

JPP, TDL, LB and GR contributed equally to this paper in various aspects of this study. KDRS carried out the isoflavone quantifications, WRC conducted the NPY analyses and EDL conceived of the study, designed, coordinated and drafted the manuscript along with the other authors.
